# Synthesis and structure of 4-{[(*E*)-(7-meth­oxy-1,3-benzodioxol-5-yl)methyl­idene]amino}-1,5-dimethyl-2-phenyl-2,3-di­hydro-1*H*-pyrazol-3-one

**DOI:** 10.1107/S2056989021000797

**Published:** 2021-01-29

**Authors:** Charmaine Arderne, Marthe Carine Djuide Fotsing, Derek Tantoh Ndinteh

**Affiliations:** a Department of Chemical Sciences, Research Centre for Synthesis and Catalysis, University of Johannesburg, PO Box 524, Auckland Park, Johannesburg, 2006, South Africa; b Department of Chemical Sciences, University of Johannesburg, PO Box 17011, Doornfontein, Johannesburg, 2028, South Africa

**Keywords:** crystal structure, Schiff bases, 4-amino­anti­pyrine, 4-amino­phenazone

## Abstract

In the title compound, C_20_H_19_N_3_O_4_, the dihedral angles between the central pyrazole ring and the pendant phenyl and substituted benzene rings are 50.95 (8) and 3.25 (12)°, respectively, and an intra­molecular C—H⋯O link generates an *S*(6) ring. The benzodioxolyl ring adopts a shallow envelope conformation with the methyl­ene C atom as the flap. In the crystal, the mol­ecules are linked by non-classical C—H⋯O inter­actions, which generate a three-dimensional network. Solvent-accessible voids run down the *c-*axis direction and the residual electron density in these voids was modelled during the refinement process using the *SQUEEZE* algorithm [Spek (2015 ▸). *Acta Cryst.* C**71**, 9–18] within the structural checking program *PLATON*.

## Abstract 2

The reaction of 4-amino-1,5-dimethyl-2-phenyl-1,2-di­hydro­pyrazol-3-one with 4-meth­oxy­benzo[1,3]dioxole-5-carbaldehyde resulted in the title compound, which crystallizes in space group *C*2/*c*. Two solvent-accessible voids, each of 397 Å^3^, were found to be evident in the crystal structure.

## Chemical context   

Compounds such as 4-amino­anti­pyrine (4-amino-1,5-dimeth­yl-2-phenyl­pyrazole) and its Schiff base analogues are chemically attractive because of the various biological properties they possess, their synthetic flexibility and their selectivity and sensitivity towards metal ions (Keskioğlu *et al.*, 2008 ▸). Pyrazol-3-one Schiff bases can be obtained from the condensation of 4-amino­phenazone or 4-amino­anti­pyrine (4-AAP) and the corresponding carbonyl compound (Sakthivel *et al.*, 2020 ▸). Schiff bases can find applications in analytical chemistry, material sciences and in various biological fields. In analytical chemistry, Schiff bases obtained from 4-AAP and 2-hydroxy-1,2-diphen­ylethenone have been used as a colorimetric sensor for Fe^III^ and as a fluorescent sensor for Al^III^ (Soufeena & Aravindakshan, 2019 ▸). Some other 4-amino­phenazone analogues have been applied in the separation and determination of penta­chloro­phenol in treated softwoods and preservative solutions (Williams, 1971 ▸). In material sciences, the corrosion inhibition tendency of 4-AAP and its derivatives has also been discussed (Junaedi *et al.*, 2013 ▸). Other derivatives have also been used to improve solar cell efficiency (Ismail *et al.*, 2020 ▸). Various 4-AAP derivatives have several biological applications and 4-AAP Schiff bases from the condensation with *para*-meth­oxy­cinnamaldehyde display anti­microbial activity against a large spectrum of microorganisms (Obasi *et al.*, 2016 ▸). Still more 4-AAP deriv­atives show DNA binding and cleavage activity has also been reported (Rosenberg *et al.*, 1969 ▸). Several other biological applications include anti­oxidant, anti-inflammatory (Deng *et al.*, 2019 ▸), analgesic and anti­pyretic (Murtaza *et al.*, 2017 ▸) among others. Platinum(II) complexes of Schiff bases have been reported as potential anti-cancer agents. Some of these complexes have a better toxicity than that of Cisplatin (Li *et al.*, 2013 ▸). 
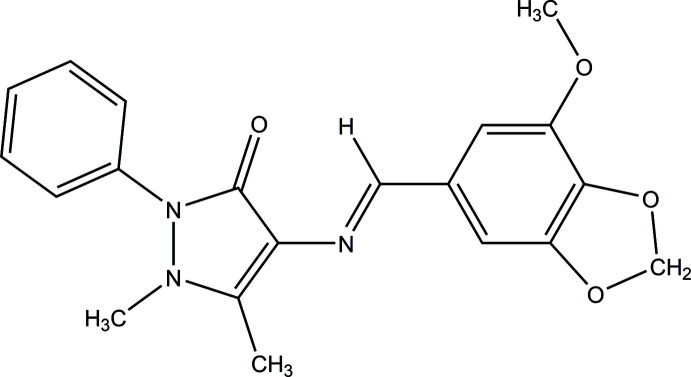


As part of our studies in this area, the title compound, C_20_H_19_N_3_O_4_, was obtained from 4-AAP and myristicin aldehyde and its crystal structure determined.

## Structural commentary   

**Figure 1:**
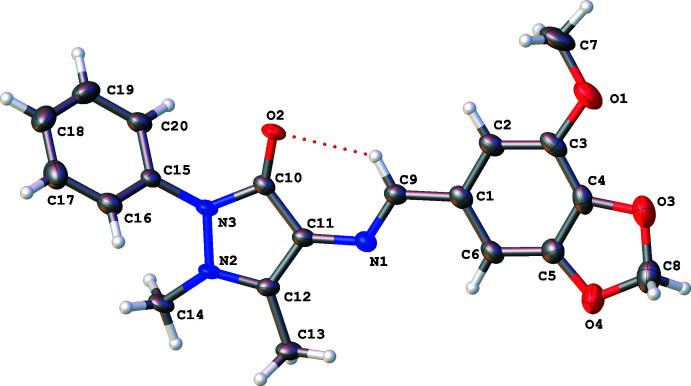
The mol­ecular structure of **I**, showing the atom-labelling scheme. Displacement ellipsoids are drawn at the 50% probability level. Dashed red lines indicate hydrogen-bonding inter­actions.

The title compound (**I**) crystallizes in the monoclinic centrosymmetric space group *C*2/*c*, and the asymmetric unit consists of one non-planar independent mol­ecule. The phenyl ring (C15–C20) is twisted away from the plane of the pyrazole ring moiety (N2/N3/C10–C12) by 50.95 (8)°, most likely because of steric hindrance of the phenyl ring and the methyl substituents on the pyrazole ring. Puckering analysis (Cremer & Pople, 1975 ▸) carried out in *PLATON* (Spek, 2020 ▸) showed that the methyl­ene carbon atom (C8) on the benzodioxolyl ring (consisting of atoms O3/C4/C5/O4/C8) can be described as the flap of an envelope with a puckering amplitude *Q* of 0.162 (2) Å and ψ angle of 323.1 (8)°. A *Mogul* (Bruno *et al.*, 2004 ▸) geometry check as performed in *Mercury* (Macrae *et al.*, 2020 ▸) did not yield any significant unusual geometrical parameters within the structure. An intra­molecular C9—H9⋯O2 hydrogen bond (Fig. 1 ▸, Table 1 ▸) generates an *S*(6) ring.

**Figure 2:**
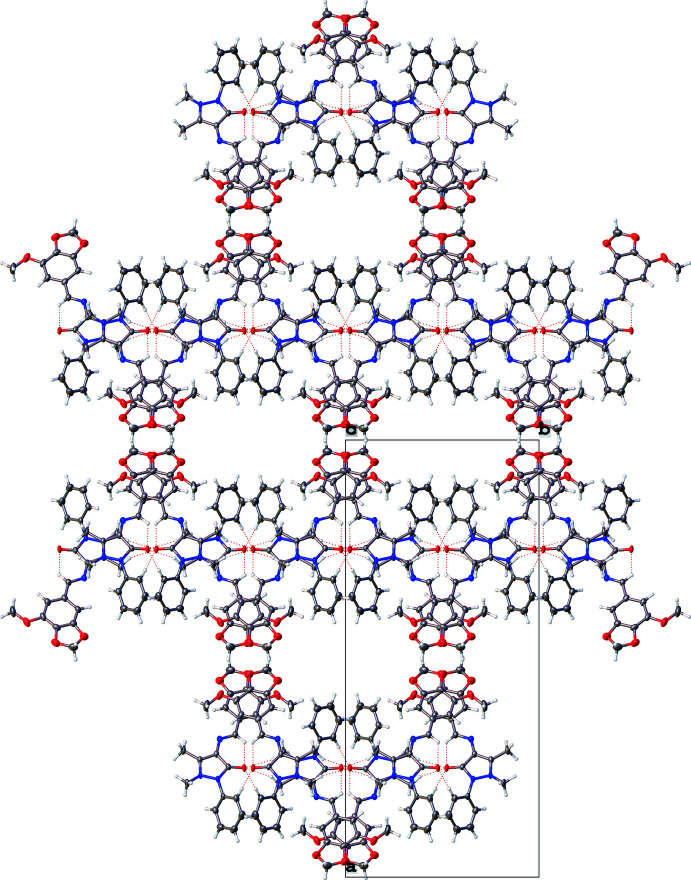
Packing diagram of **I** as viewed down the *c*-axis direction. Dashed red lines indicate hydrogen-bonding inter­actions.

Inter­estingly, after completing the structural refinement the structural checks suggested that the structure contains two solvent-accessible voids, each of 397 Å^3^. The *PLATON* SQUEEZE (Spek, 2015 ▸) algorithm was applied to the refinement to explain this structural feature and assign the electron density accordingly. Since the material was synthesized in ethanol, it is likely that the voids were created by the solvent and once the crystals were extracted from the reaction mixture and the solvent evaporated, voids were formed in this way. The voids can be seen in the packing arrangement (Fig. 2 ▸).

## Supra­molecular features   

**Figure 3:**
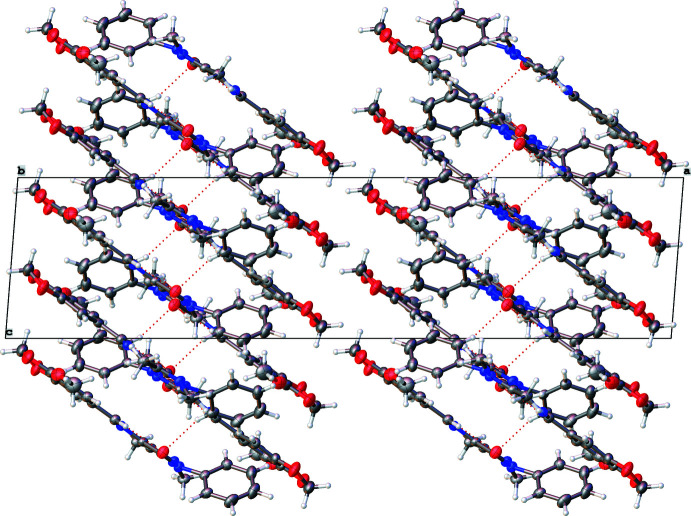
Packing diagram of **I** as viewed down the *b*-axis direction. Dashed red lines indicate hydrogen-bonding inter­actions.

**Figure 4:**
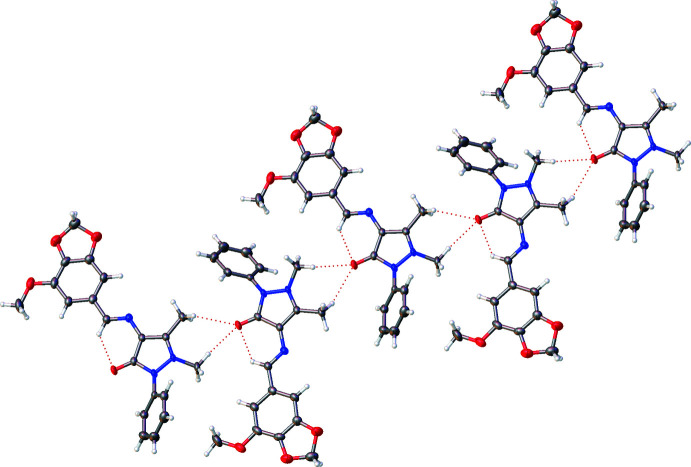
Detail of the structure of **I** showing three of the four hydrogen-bonding inter­actions; one intra­molecular inter­action and two of the three inter­molecular inter­actions are indicated by dashed red lines.

Analysis of the crystal packing of **I** clearly shows the channels of void space, especially when viewed down the *c-*axis direction (Fig. 2 ▸). The mol­ecules tend to stack on top of one another in an alternate fashion, as is evident when viewed down the *b-*axis direction (Fig. 3 ▸) with the phenyl rings protruding out of the plane every alternate layer. While there are no classical hydrogen bonds, there are hydrogen-bonding inter­actions present (mostly C—H⋯O inter­actions; Table 1 ▸), which help to consolidate the packing. This is particularly evident in Fig. 3 ▸ where the hydrogen bonds can be seen to be connecting layers of mol­ecules together. The hydrogen-bonding network (three-dimensional in nature) showing the four most prominent hydrogen-bonding inter­actions (one being an intra­molecular inter­action) can be seen in Fig. 4 ▸. It may be noted that atom O2 accepts all the hydrogen bonds (one intra­molecular and three inter­molecular). The second graph-set that is clearly visible in Fig. 4 ▸ is a ring motif with graph-set descriptor 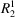
(7). It is these inter­molecular inter­actions that connect the mol­ecules between layers, as shown in Fig. 3 ▸. Two weak C—H⋯π inter­actions are also present (Table 1 ▸).

## Database survey   

A search for the exact structure of the title compound in the Cambridge Structural Database (CSD Version 2020.2.0; Groom *et al.*, 2016 ▸) yielded no hits. In order to determine if the structures of other similar compounds had been published, we expanded the structure search to only include the 2,3-*D*i­hydro-1*H*-pyrazole moiety as the backbone for other possible structures. A search was carried out in the CSD with no filters applied and this yielded 322 compounds. Of these, 92 of the compounds were coordinated to metals or were co-crystals and classified as ‘organometallic? under the CSD search filter. The remaining 230 compounds are then classified as ‘organic? under the CSD search filter. Thus, the title compound falls into this latter category.

## Synthesis and crystallization   

The title compound was prepared by reflux of a solution containing 4-amino-1,5-dimethyl-2-phenyl-1,2-di­hydro­pyrazol-3-one (0.244 g, 1.20 mmol) in 5 ml of ethanol and a solution of 4-meth­oxy­benzo[1,3]dioxole-5-carbaldehyde (0.179 g, 1.20 mmol) in 5 ml of ethanol. The reaction mixture was stirred for 24 h under reflux. Crystals of the title compound were obtained from ethanol solution by slow evaporation. A suitable crystal was selected from the mother liquor for the single-crystal X-ray diffraction analysis.

## Refinement   

Crystal data, data collection and structure refinement details are summarized in Table 2 ▸. The C-bound H atoms were placed in geometrically idealized positions, with C—H = 0.93–0.99 Å, and were constrained to ride on their parent atoms with relative isotropic displacement coefficients, with *U*_iso_(H) = 1.2*U*_eq_(C) for aromatic and methyl­ene H atoms, and *U*_iso_(H) = 1.5*U*_eq_(C) for methyl H atoms. The methyl H atoms were initially located in a different-Fourier map and they were placed in idealized positions as described above and refined as rotating groups. The structure contained two solvent accessible voids of 397 Å^3^ each, thereby giving a total void volume of 794 Å^3^. No substantial electron density peaks were found in the solvent-accessible voids and the residual electron density peaks could not arranged in an inter­pretable pattern. The cif and fcf files were thus corrected for using reverse Fourier transform methods using the *SQUEEZE* routine (Spek, 2015 ▸) as implemented in the program *PLATON* (Spek, 2020 ▸). The resultant files were used in the further refinement. The *SQUEEZE* procedure corrected for 28 electrons within the two solvent-accessible voids.

## Supplementary Material

Crystal structure: contains datablock(s) I. DOI: 10.1107/S2056989021000797/hb7958sup1.cif

Structure factors: contains datablock(s) I. DOI: 10.1107/S2056989021000797/hb7958Isup2.hkl

Supporting information file. DOI: 10.1107/S2056989021000797/hb7958Isup3.mol

Supporting information file. DOI: 10.1107/S2056989021000797/hb7958Isup4.cml

CCDC reference: 2058001

Additional supporting information:  crystallographic information; 3D view; checkCIF report

## Figures and Tables

**Table 1:** Hydrogen-bond geometry (Å, °) *Cg*2 and *Cg*4 are the centroids of the pyrazole (N2/N3/C10–C12) and phenyl (C15–C20) rings, respectively.

*D*—H⋯*A*	*D*—H	H⋯*A*	*D*⋯*A*	*D*—H⋯*A*
C9—H9⋯O2	0.95	2.33	3.031 (2)	131
C13—H13*A*⋯O2^i^	0.98	2.62	3.265 (2)	124
C14—H14*B*⋯O2^i^	0.98	2.38	3.330 (2)	163
C20—H20⋯O2^ii^	0.95	2.57	3.488 (3)	162
C14—H14*C*⋯*Cg*2^iii^	0.98	2.72	3.584 (3)	147
C19—H19⋯*Cg*4^ii^	0.95	2.94	3.816 (3)	154

Symmetry codes: (i) 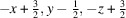; (ii) 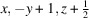; (iii) 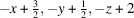.

**Table 2:** Experimental details

Crystal data	
Chemical formula	C_20_H_19_N_3_O_4_
*M*_r_	365.38
Crystal system, space group	Monoclinic, *C*2/*c*
Temperature (K)	173
*a*, *b*, *c* (Å)	33.888 (4), 14.9497 (18), 8.2021 (10)
β (°)	94.447 (4)
*V* (Å^3^)	4142.8 (9)
*Z*	8
Radiation type	Mo *K*α
μ (mm^−1^)	0.08
Crystal size (mm)	0.43 × 0.37 × 0.03

Data collection	
Diffractometer	Bruker APEXII CCD
Absorption correction	Multi-scan (*SADABS*; Bruker, 2016 ▸)
*T*_min_, *T*_max_	0.961, 0.969
No. of measured, independent and observed [*I* > 2σ(*I*)] reflections	16905, 5003, 2935
*R*_int_	0.068
(sin θ/λ)_max_ (Å^−1^)	0.660

Refinement	
*R*[*F* ^2^ > 2σ(*F* ^2^)], *wR*(*F* ^2^), *S*	0.063, 0.161, 1.05
No. of reflections	5003
No. of parameters	247
H-atom treatment	H-atom parameters constrained
Δρ_max_, Δρ_min_ (e Å^−3^)	0.22, −0.25

Computer programs: *APEX2* (Bruker, 2014 ▸), *SAINT* (Bruker, 2015 ▸), *SHELXL2018/3* (Sheldrick, 2015 ▸), *OLEX2* (Dolomanov *et al.*, 2009 ▸).

## supplementary crystallographic information

### Crystal data

**Table:**

C_20_H_19_N_3_O_4_	*F*(000) = 1536
*M**_r_* = 365.38	*D*_x_ = 1.172 Mg m^−^^3^
Monoclinic, *C*2/*c*	Mo *K*α radiation, λ = 0.71073 Å
*a* = 33.888 (4) Å	Cell parameters from 4074 reflections
*b* = 14.9497 (18) Å	θ = 2.9–28.0°
*c* = 8.2021 (10) Å	µ = 0.08 mm^−^^1^
β = 94.447 (4)°	*T* = 173 K
*V* = 4142.8 (9) Å^3^	Plate, colourless
*Z* = 8	0.43 × 0.37 × 0.03 mm

### Data collection

**Table:**

Bruker APEXII CCD diffractometer	2935 reflections with *I* > 2σ(*I*)
φ and ω scans	*R*_int_ = 0.068
Absorption correction: multi-scan (SADABS; Bruker, 2016)	θ_max_ = 28.0°, θ_min_ = 2.9°
*T*_min_ = 0.961, *T*_max_ = 0.969	*h* = −44→44
16905 measured reflections	*k* = −19→19
5003 independent reflections	*l* = −10→10

### Refinement

**Table:**

Refinement on *F*^2^	Primary atom site location: dual
Least-squares matrix: full	Hydrogen site location: inferred from neighbouring sites
*R*[*F*^2^ > 2σ(*F*^2^)] = 0.063	H-atom parameters constrained
*wR*(*F*^2^) = 0.161	*w* = 1/[σ^2^(*F*_o_^2^) + (0.0734*P*)^2^ + 0.941*P*] where *P* = (*F*_o_^2^ + 2*F*_c_^2^)/3
*S* = 1.05	(Δ/σ)_max_ = 0.001
5003 reflections	Δρ_max_ = 0.22 e Å^−^^3^
247 parameters	Δρ_min_ = −0.24 e Å^−^^3^
0 restraints	

### Special details

**Table:**

Geometry. All esds (except the esd in the dihedral angle between two l.s. planes) are estimated using the full covariance matrix. The cell esds are taken into account individually in the estimation of esds in distances, angles and torsion angles; correlations between esds in cell parameters are only used when they are defined by crystal symmetry. An approximate (isotropic) treatment of cell esds is used for estimating esds involving l.s. planes.

### Fractional atomic coordinates and isotropic or equivalent isotropic displacement parameters (Å^2^)

**Table:**

	*x*	*y*	*z*	*U*_iso_*/*U*_eq_	
O1	0.58334 (5)	0.64735 (10)	0.2240 (2)	0.0456 (4)	
O2	0.75013 (4)	0.47729 (8)	0.70830 (18)	0.0323 (4)	
O3	0.53604 (5)	0.49708 (11)	0.1150 (2)	0.0494 (5)	
O4	0.55059 (4)	0.34999 (10)	0.1851 (2)	0.0503 (5)	
N1	0.68411 (5)	0.35046 (10)	0.54319 (19)	0.0252 (4)	
N2	0.76699 (5)	0.25079 (9)	0.7655 (2)	0.0249 (4)	
N3	0.77585 (5)	0.34100 (9)	0.7990 (2)	0.0255 (4)	
C1	0.63820 (6)	0.45058 (13)	0.4033 (3)	0.0279 (5)	
C2	0.62927 (6)	0.53913 (13)	0.3626 (3)	0.0312 (5)	
H2	0.646875	0.585052	0.402084	0.037*	
C3	0.59537 (7)	0.56216 (13)	0.2659 (3)	0.0340 (5)	
C4	0.57152 (6)	0.49285 (15)	0.2094 (3)	0.0340 (5)	
C5	0.58021 (6)	0.40553 (14)	0.2506 (3)	0.0327 (5)	
C6	0.61272 (6)	0.38165 (14)	0.3489 (3)	0.0312 (5)	
H6	0.617801	0.321120	0.378844	0.037*	
C7	0.60737 (8)	0.71811 (15)	0.2899 (4)	0.0576 (8)	
H7A	0.595674	0.775502	0.254306	0.086*	
H7B	0.633927	0.712931	0.251285	0.086*	
H7C	0.609129	0.714853	0.409539	0.086*	
C8	0.52643 (7)	0.40623 (16)	0.0732 (3)	0.0467 (6)	
H8A	0.532170	0.393770	−0.041099	0.056*	
H8B	0.497994	0.394756	0.084311	0.056*	
C9	0.67425 (6)	0.43094 (12)	0.5059 (2)	0.0277 (5)	
H9	0.690725	0.478813	0.545809	0.033*	
C10	0.74742 (5)	0.39514 (12)	0.7154 (2)	0.0238 (4)	
C11	0.71847 (6)	0.33316 (12)	0.6432 (2)	0.0237 (4)	
C12	0.73153 (6)	0.24798 (12)	0.6818 (2)	0.0243 (4)	
C13	0.71041 (6)	0.16214 (12)	0.6430 (3)	0.0306 (5)	
H13A	0.729441	0.116956	0.612385	0.046*	
H13B	0.690370	0.171445	0.551884	0.046*	
H13C	0.697547	0.141629	0.739228	0.046*	
C14	0.78468 (6)	0.18201 (12)	0.8739 (3)	0.0299 (5)	
H14A	0.811992	0.171173	0.847614	0.045*	
H14B	0.769373	0.126576	0.859300	0.045*	
H14C	0.784562	0.202030	0.987620	0.045*	
C15	0.81520 (6)	0.36838 (12)	0.8489 (2)	0.0239 (4)	
C16	0.84730 (6)	0.32824 (13)	0.7854 (3)	0.0328 (5)	
H16	0.843312	0.281952	0.706444	0.039*	
C17	0.88527 (7)	0.35536 (15)	0.8364 (3)	0.0430 (6)	
H17	0.907443	0.327095	0.794364	0.052*	
C18	0.89074 (7)	0.42389 (15)	0.9491 (3)	0.0501 (7)	
H18	0.916768	0.442702	0.984695	0.060*	
C19	0.85839 (7)	0.46524 (15)	1.0102 (3)	0.0470 (6)	
H19	0.862273	0.513220	1.085733	0.056*	
C20	0.82043 (7)	0.43677 (13)	0.9616 (3)	0.0328 (5)	
H20	0.798210	0.464009	1.005186	0.039*	

### Atomic displacement parameters (Å^2^)

**Table:**

	*U*^11^	*U*^22^	*U*^33^	*U*^12^	*U*^13^	*U*^23^
O1	0.0564 (11)	0.0334 (9)	0.0463 (10)	0.0173 (8)	−0.0007 (8)	0.0064 (7)
O2	0.0376 (9)	0.0122 (7)	0.0458 (10)	0.0028 (6)	−0.0055 (7)	−0.0010 (6)
O3	0.0397 (10)	0.0505 (11)	0.0560 (12)	0.0124 (8)	−0.0105 (8)	0.0039 (8)
O4	0.0374 (9)	0.0456 (10)	0.0646 (12)	0.0024 (8)	−0.0170 (8)	0.0046 (8)
N1	0.0277 (9)	0.0207 (8)	0.0271 (10)	0.0021 (7)	0.0011 (7)	0.0018 (7)
N2	0.0330 (10)	0.0104 (8)	0.0303 (10)	0.0012 (7)	−0.0037 (7)	0.0008 (7)
N3	0.0304 (9)	0.0126 (8)	0.0327 (10)	0.0015 (7)	−0.0023 (7)	−0.0003 (7)
C1	0.0322 (11)	0.0269 (11)	0.0250 (11)	0.0068 (9)	0.0044 (9)	−0.0002 (8)
C2	0.0389 (12)	0.0241 (11)	0.0305 (12)	0.0062 (9)	0.0019 (10)	0.0004 (9)
C3	0.0440 (13)	0.0270 (11)	0.0316 (13)	0.0136 (10)	0.0072 (10)	0.0051 (9)
C4	0.0290 (12)	0.0429 (13)	0.0297 (13)	0.0119 (10)	−0.0009 (10)	0.0036 (10)
C5	0.0275 (11)	0.0328 (12)	0.0378 (13)	0.0024 (9)	0.0024 (10)	−0.0017 (10)
C6	0.0323 (12)	0.0248 (11)	0.0361 (13)	0.0085 (9)	0.0011 (10)	0.0012 (9)
C7	0.0775 (19)	0.0230 (12)	0.072 (2)	0.0129 (13)	0.0009 (16)	0.0026 (12)
C8	0.0350 (13)	0.0563 (16)	0.0472 (16)	0.0013 (12)	−0.0059 (11)	0.0091 (12)
C9	0.0320 (11)	0.0213 (10)	0.0295 (12)	0.0017 (8)	0.0000 (9)	−0.0006 (8)
C10	0.0273 (11)	0.0189 (10)	0.0253 (11)	0.0041 (8)	0.0027 (8)	0.0016 (8)
C11	0.0307 (11)	0.0173 (9)	0.0228 (11)	0.0004 (8)	0.0005 (9)	−0.0004 (8)
C12	0.0316 (11)	0.0177 (9)	0.0231 (11)	−0.0008 (8)	−0.0002 (9)	−0.0007 (8)
C13	0.0417 (12)	0.0166 (9)	0.0328 (12)	−0.0037 (9)	−0.0011 (10)	0.0030 (8)
C14	0.0438 (13)	0.0146 (9)	0.0306 (12)	0.0041 (9)	−0.0022 (10)	0.0051 (8)
C15	0.0288 (11)	0.0153 (9)	0.0265 (11)	0.0022 (8)	−0.0044 (9)	0.0034 (8)
C16	0.0379 (13)	0.0243 (10)	0.0359 (13)	0.0049 (9)	0.0008 (10)	0.0001 (9)
C17	0.0308 (12)	0.0394 (13)	0.0582 (17)	0.0023 (10)	0.0003 (11)	0.0085 (12)
C18	0.0396 (14)	0.0363 (13)	0.0710 (19)	−0.0072 (11)	−0.0175 (13)	0.0039 (13)
C19	0.0533 (16)	0.0303 (12)	0.0539 (17)	−0.0060 (11)	−0.0179 (13)	−0.0055 (11)
C20	0.0421 (13)	0.0218 (10)	0.0332 (13)	0.0053 (9)	−0.0055 (10)	−0.0020 (9)

### Geometric parameters (Å, º)

**Table:**

O1—C3	1.373 (2)	C7—H7C	0.9800
O1—C7	1.416 (3)	C8—H8A	0.9900
O2—C10	1.233 (2)	C8—H8B	0.9900
O3—C4	1.380 (3)	C9—H9	0.9500
O3—C8	1.432 (3)	C10—C11	1.443 (3)
O4—C5	1.379 (2)	C11—C12	1.377 (2)
O4—C8	1.450 (3)	C12—C13	1.492 (3)
N1—C9	1.279 (2)	C13—H13A	0.9800
N1—C11	1.396 (2)	C13—H13B	0.9800
N2—N3	1.404 (2)	C13—H13C	0.9800
N2—C12	1.337 (2)	C14—H14A	0.9800
N2—C14	1.458 (2)	C14—H14B	0.9800
N3—C10	1.397 (2)	C14—H14C	0.9800
N3—C15	1.424 (2)	C15—C16	1.379 (3)
C1—C2	1.393 (3)	C15—C20	1.380 (3)
C1—C6	1.395 (3)	C16—H16	0.9500
C1—C9	1.459 (3)	C16—C17	1.383 (3)
C2—H2	0.9500	C17—H17	0.9500
C2—C3	1.388 (3)	C17—C18	1.382 (3)
C3—C4	1.372 (3)	C18—H18	0.9500
C4—C5	1.374 (3)	C18—C19	1.386 (3)
C5—C6	1.361 (3)	C19—H19	0.9500
C6—H6	0.9500	C19—C20	1.384 (3)
C7—H7A	0.9800	C20—H20	0.9500
C7—H7B	0.9800		
			
C3—O1—C7	116.55 (18)	N1—C9—H9	119.4
C4—O3—C8	105.24 (17)	C1—C9—H9	119.4
C5—O4—C8	104.81 (16)	O2—C10—N3	123.30 (17)
C9—N1—C11	120.35 (17)	O2—C10—C11	132.15 (17)
N3—N2—C14	119.11 (15)	N3—C10—C11	104.50 (15)
C12—N2—N3	107.50 (14)	N1—C11—C10	129.19 (16)
C12—N2—C14	126.98 (16)	C12—C11—N1	123.04 (17)
N2—N3—C15	120.91 (15)	C12—C11—C10	107.67 (17)
C10—N3—N2	109.35 (15)	N2—C12—C11	110.41 (16)
C10—N3—C15	124.72 (15)	N2—C12—C13	122.25 (17)
C2—C1—C6	120.48 (19)	C11—C12—C13	127.32 (18)
C2—C1—C9	119.11 (19)	C12—C13—H13A	109.5
C6—C1—C9	120.40 (18)	C12—C13—H13B	109.5
C1—C2—H2	119.1	C12—C13—H13C	109.5
C3—C2—C1	121.9 (2)	H13A—C13—H13B	109.5
C3—C2—H2	119.1	H13A—C13—H13C	109.5
O1—C3—C2	126.1 (2)	H13B—C13—H13C	109.5
C4—C3—O1	117.4 (2)	N2—C14—H14A	109.5
C4—C3—C2	116.44 (19)	N2—C14—H14B	109.5
C3—C4—O3	128.3 (2)	N2—C14—H14C	109.5
C3—C4—C5	121.7 (2)	H14A—C14—H14B	109.5
C5—C4—O3	110.0 (2)	H14A—C14—H14C	109.5
C4—C5—O4	109.91 (18)	H14B—C14—H14C	109.5
C6—C5—O4	127.15 (19)	C16—C15—N3	120.95 (17)
C6—C5—C4	122.9 (2)	C16—C15—C20	120.75 (19)
C1—C6—H6	121.7	C20—C15—N3	118.30 (18)
C5—C6—C1	116.63 (19)	C15—C16—H16	120.0
C5—C6—H6	121.7	C15—C16—C17	120.0 (2)
O1—C7—H7A	109.5	C17—C16—H16	120.0
O1—C7—H7B	109.5	C16—C17—H17	120.2
O1—C7—H7C	109.5	C18—C17—C16	119.6 (2)
H7A—C7—H7B	109.5	C18—C17—H17	120.2
H7A—C7—H7C	109.5	C17—C18—H18	119.9
H7B—C7—H7C	109.5	C17—C18—C19	120.2 (2)
O3—C8—O4	106.97 (18)	C19—C18—H18	119.9
O3—C8—H8A	110.3	C18—C19—H19	119.9
O3—C8—H8B	110.3	C20—C19—C18	120.1 (2)
O4—C8—H8A	110.3	C20—C19—H19	119.9
O4—C8—H8B	110.3	C15—C20—C19	119.3 (2)
H8A—C8—H8B	108.6	C15—C20—H20	120.4
N1—C9—C1	121.24 (18)	C19—C20—H20	120.4
			
O1—C3—C4—O3	−0.6 (3)	C6—C1—C9—N1	2.6 (3)
O1—C3—C4—C5	−177.19 (19)	C7—O1—C3—C2	−1.8 (3)
O2—C10—C11—N1	−0.8 (4)	C7—O1—C3—C4	177.1 (2)
O2—C10—C11—C12	175.6 (2)	C8—O3—C4—C3	172.5 (2)
O3—C4—C5—O4	−0.3 (2)	C8—O3—C4—C5	−10.5 (2)
O3—C4—C5—C6	−177.30 (19)	C8—O4—C5—C4	10.8 (2)
O4—C5—C6—C1	−178.5 (2)	C8—O4—C5—C6	−172.3 (2)
N1—C11—C12—N2	173.82 (17)	C9—N1—C11—C10	−1.2 (3)
N1—C11—C12—C13	−7.4 (3)	C9—N1—C11—C12	−177.11 (18)
N2—N3—C10—O2	−171.96 (17)	C9—C1—C2—C3	−179.79 (19)
N2—N3—C10—C11	5.89 (19)	C9—C1—C6—C5	−178.54 (19)
N2—N3—C15—C16	36.3 (3)	C10—N3—C15—C16	−115.9 (2)
N2—N3—C15—C20	−144.29 (18)	C10—N3—C15—C20	63.5 (3)
N3—N2—C12—C11	6.5 (2)	C10—C11—C12—N2	−2.8 (2)
N3—N2—C12—C13	−172.36 (16)	C10—C11—C12—C13	175.95 (18)
N3—C10—C11—N1	−178.37 (18)	C11—N1—C9—C1	−179.18 (17)
N3—C10—C11—C12	−2.0 (2)	C12—N2—N3—C10	−7.8 (2)
N3—C15—C16—C17	−179.51 (18)	C12—N2—N3—C15	−163.85 (17)
N3—C15—C20—C19	−179.17 (19)	C14—N2—N3—C10	−161.64 (16)
C1—C2—C3—O1	177.54 (19)	C14—N2—N3—C15	42.3 (2)
C1—C2—C3—C4	−1.4 (3)	C14—N2—C12—C11	157.66 (18)
C2—C1—C6—C5	2.4 (3)	C14—N2—C12—C13	−21.2 (3)
C2—C1—C9—N1	−178.33 (19)	C15—N3—C10—O2	−17.0 (3)
C2—C3—C4—O3	178.5 (2)	C15—N3—C10—C11	160.81 (18)
C2—C3—C4—C5	1.9 (3)	C15—C16—C17—C18	−1.2 (3)
C3—C4—C5—O4	176.88 (19)	C16—C15—C20—C19	0.2 (3)
C3—C4—C5—C6	−0.1 (3)	C16—C17—C18—C19	−0.1 (4)
C4—O3—C8—O4	16.9 (2)	C17—C18—C19—C20	1.4 (4)
C4—C5—C6—C1	−2.1 (3)	C18—C19—C20—C15	−1.5 (3)
C5—O4—C8—O3	−17.1 (2)	C20—C15—C16—C17	1.1 (3)
C6—C1—C2—C3	−0.8 (3)		

### Hydrogen-bond geometry (Å, º)

*Cg*2 and *Cg*4 are the centroids of the pyrazole (N2/N3/C10–C12) and phenyl (C15–C20) rings, respectively.

**Table:**

*D*—H···*A*	*D*—H	H···*A*	*D*···*A*	*D*—H···*A*
C9—H9···O2	0.95	2.33	3.031 (2)	131
C13—H13*A*···O2^i^	0.98	2.62	3.265 (2)	124
C14—H14*B*···O2^i^	0.98	2.38	3.330 (2)	163
C20—H20···O2^ii^	0.95	2.57	3.488 (3)	162
C14—H14*C*···*Cg*2^iii^	0.98	2.72	3.584 (3)	147
C19—H19···*Cg*4^ii^	0.95	2.94	3.816 (3)	154

Symmetry codes: (i) −*x*+3/2, *y*−1/2, −*z*+3/2; (ii) *x*, −*y*+1, *z*+1/2; (iii) −*x*+3/2, −*y*+1/2, −*z*+2.
